# Retroperitoneal ganglioneuroma with nodal involvement in an adult patient with human immunodeficiency virus: a case report and review of the literature

**DOI:** 10.1186/s13256-021-03134-4

**Published:** 2021-12-28

**Authors:** Elliott Lebby, Daniel Kwan, Thanh-Lan Bui, Ryan O’Connell, Mani Seetharaman, Roozbeh Houshyar

**Affiliations:** 1 Irvine Department of Radiological Sciences, University of California, 101 The City Drive South, Route 140, Orange, CA 92868 USA; 2 Irvine Department of Pathology. UCI School of Medicine D440 Medical Sciences I, University of California, Irvine, CA 92697 USA

**Keywords:** Ganglioneuroma, Diagnostic imaging, Case report

## Abstract

**Background:**

Ganglioneuromas are a benign tumor originating from neural crest cells. As one of the neuroblastic tumors, ganglioneuromas are most common in children, with a mean age at presentation of 7 years. Ganglioneuromas are typically singular in nature, but rarely can present with lymph node involvement and distant metastasis. We present a rare case of a retroperitoneal ganglioneuroma found in a human immunodeficiency virus positive adult, which was complicated by lymph node involvement. This case is notable not only in regard to the age of the patient, but also because of his human immunodeficiency virus positive status and the extension of tumor to lymph nodes.

**Case presentation:**

A 27-year-old Latino male with history of human immunodeficiency virus presented with a 6-month history of left upper quadrant and epigastric abdominal pain with associated nausea and vomiting. The patient had a computed tomography scan showing a retroperitoneal mass encasing the aorta, celiac axis, and superior mesenteric artery. Core needle biopsy revealed ganglioneuroma. Owing to obstructive symptoms, resection of the mass along with partial gastric resection, partial pancreatic resection, and splenectomy was performed by a multidisciplinary group of surgeons. Pathology results revealed metastatic spread of ganglioneuroma to surrounding lymph nodes.

**Conclusions:**

Ganglioneuromas are most common in children and very rarely occur in adults. However, it is still important to consider this entity in the differential for patients presenting with suspicious symptoms. While rare, it is essential to consider metastasis in this generally benign disease.

## Background

Ganglioneuromas are benign tumors of neural crest cells. Along with ganglioneuroblastomas and neuroblastomas, they make up the neuroblastic tumors. Ganglioneuromas may form either spontaneously or through maturation of another, more immature, neuroblastic tumor [1]. They present most commonly in children, with a reported mean age at presentation of 7 years [2–4]. Ganglioneuromas are most often discovered incidentally; however, non-specific symptoms including abdominal pain and dyspnea may be present [4, 5]. Imaging with computed tomography (CT) or magnetic resonance imaging (MRI) can aid in detection. Definitive diagnosis requires histologic analysis. While the tumor is usually singular in nature, there are a few reported cases of lymph node involvement and distant metastasis [3, 6]. Resection is generally curative, and prognosis is excellent [4, 5]. Here, we present a rare case of a human immunodeficiency virus (HIV) positive adult with retroperitoneal ganglioneuroma complicated by lymph node involvement. To our knowledge, there are no previously published reports of an HIV positive adult with ganglioneuroma that was complicated by metastasis.

## Case presentation

A 27-year-old Latino male with HIV presented to the emergency department complaining of a 6-month history of constant, non-radiating, left upper quadrant abdominal and epigastric pain, with associated nausea and vomiting. Besides HIV, the patient’s past medical and surgical history was unremarkable. The exact date of HIV diagnosis was not available in the patient’s medical record, but at the time of presentation, the patient was not on antiretroviral medication. Social and environmental history were remarkable for a 2-pack-year history of tobacco use and less than one alcoholic drink per week. His family history was only notable for diabetes and hypertension in several relatives. Physical examination revealed diffuse abdominal tenderness. Respiratory and cardiovascular examinations were within normal limits. There were no skin lesions. Neurologic examination revealed no focal neurological deficits. Vitals were notable for mild tachycardia to 105 beats per minute. Temperature was 37°C and blood pressure was 124/64 mmHg. The patient had no significant abnormal lab findings other than an elevated total protein of 9.4 g/dL (6.0–8.3 g/dL), elevated total bilirubin of 1.5 mg/dL (< 1.2 mg/dL), and high HIV viral load of greater than 100,000 copies/mL. Complete blood count, basic metabolic panel, and transaminases were normal at presentation.

**Fig. 1:**
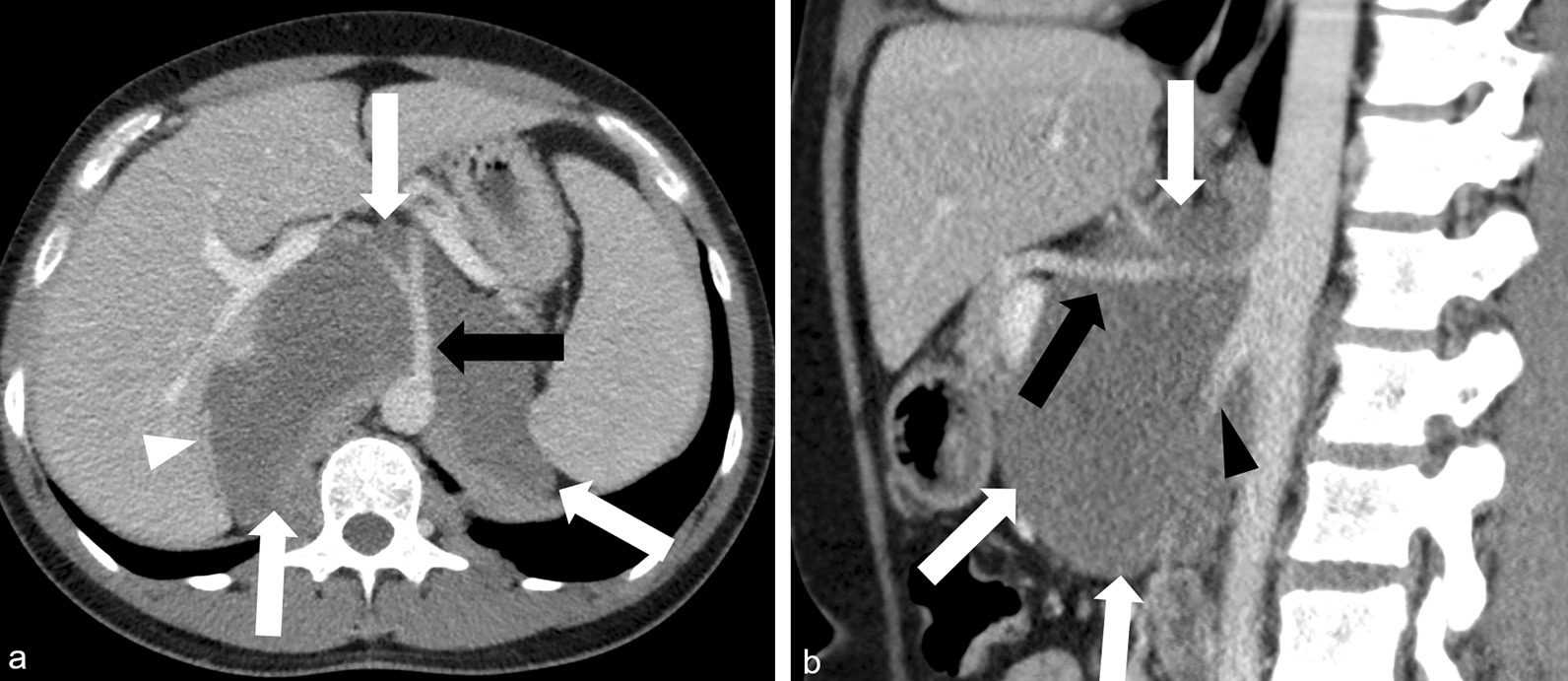
Axial (**a**) and sagittal (**b**) contrast-enhanced computed tomography of the abdomen demonstrates a large well-circumscribed hypodense mass in the retroperitoneum (white arrows), with involvement of the right adrenal gland (white arrowhead), encasement of the celiac axis and its branches (black arrows), and encasement of the superior mesenteric artery (black arrowhead)

Contrast-enhanced computed tomography (CT) of the chest, abdomen, and pelvis (Fig. 1) at initial presentation demonstrated a large hypodense retroperitoneal mass encasing the aorta, celiac axis, superior mesenteric artery (SMA), and right adrenal gland, with associated mass effect on adjacent organs and vessels. Additionally, splenomegaly and a small right anterior chest wall subcutaneous soft tissue nodule were noted. Core needle biopsy of the retroperitoneal mass performed 2 weeks after presentation demonstrated Schwannian stroma and scattered mature ganglion cells. The tumor stained strongly positive for S100, consistent with ganglioneuroma.

During his stay, the patient was started on 1 elvitegravir-cobicistat-emtricitabine-tenofovir tablet daily for HIV, melatonin 5 mg tablet nightly as a sleep aid, acetaminophen 650 mg tablet every 4 hours for pain, and ondansetron 4 mg tablet every 8 hours for nausea as needed. His antiretroviral medication was continued at discharge.

**Fig. 2:**
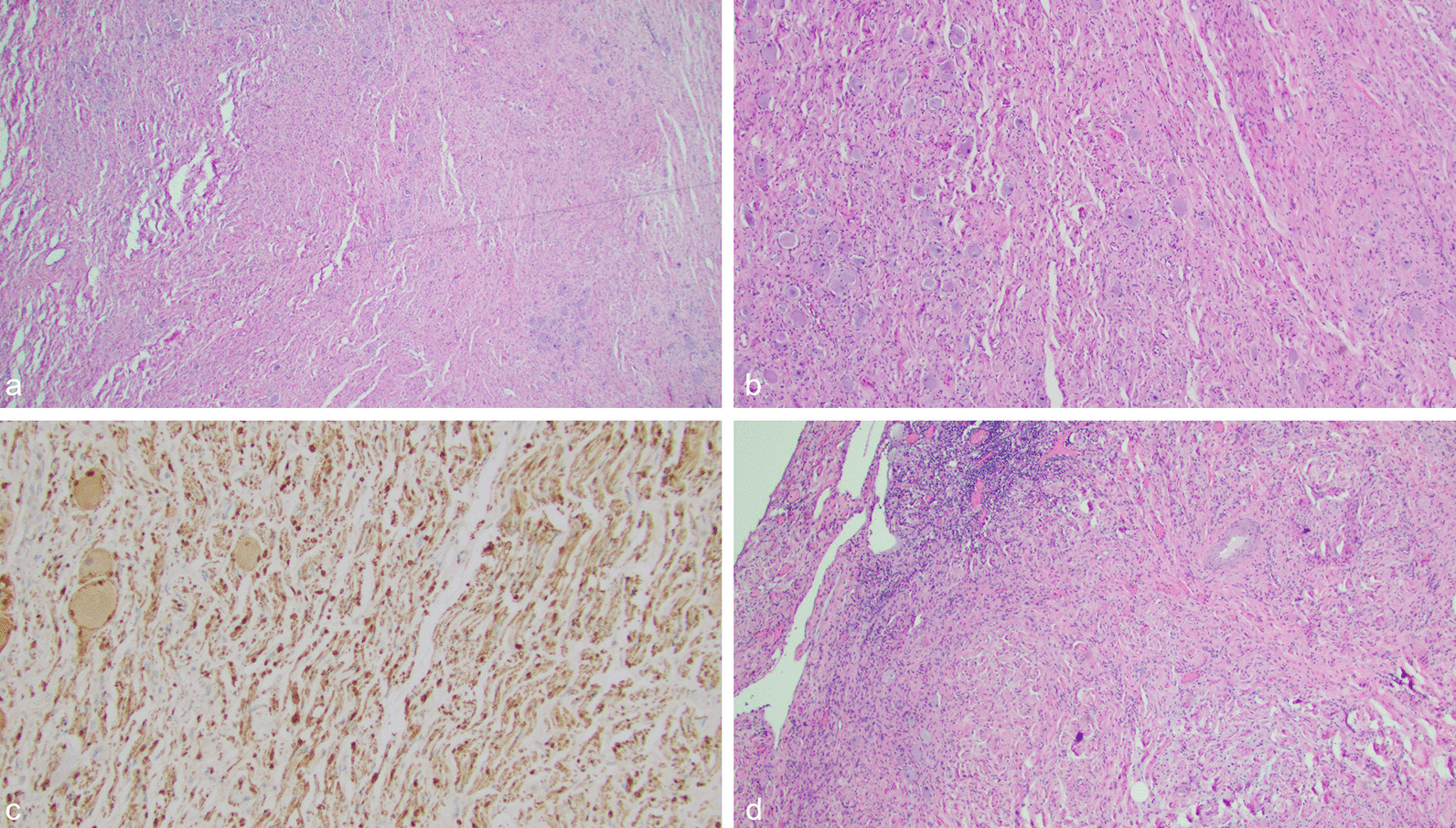
Images show a Schwannian stroma dominant ganglioneuroma, maturing subtype, with favorable histology. This tumor likely represents a biologically favorable neuroblastoma that metastasized to the lymph nodes early in the clinical course and subsequently showed ganglioneuromatous maturation. **a** Hematoxylin and eosin (H&E) stain at 20× magnification showing a Schwannian stroma dominant tumor with mature ganglion cells with abundant dense eosinophilic cytoplasm, eccentric nuclei, and prominent nucleoli. **b** H&E stain at 40× magnification showing Schwannian stroma dominant tumor with mature ganglion cells with abundant dense eosinophilic cytoplasm, eccentric nuclei, and prominent nucleoli. **c** S100 immunohistochemical stain at 100× magnification showing strong positive staining within the Schwannian stroma dominant tumor. **d** H&E stain at 40× magnification showing ganglioneuroma metastatic to a lymph node

After multidisciplinary review, chemotherapy and radiation were not recommended. Because of extensive vascular encasement, the tumor was initially deemed to be unresectable; however, after deliberation, the decision was made to attempt resection as it was the only viable therapy. Three months after presentation, colorectal, vascular, and hepatobiliary surgery performed an exploratory laparoscopy and eventual resection of the mass, along with a partial gastric resection, partial pancreatic resection, right adrenalectomy, periportal lymphadenectomy, and splenectomy. The tumor was indistinguishable from the adrenal gland, which was its likely origin. Histology of the resected lymph nodes showed metastatic ganglioneuroma, which was determined to be clinically insignificant because of favorable histology (Fig. 2). Immediate post-operative recovery was uneventful, and the surgery was curative.

In the years following surgery, the patient experienced chronic diarrhea and frequent lower abdominal pain without a discernible cause despite extensive gastrointestinal workup.

## Discussion

Here, we presented the case of a retroperitoneal ganglioneuroma with lymph node involvement in an adult patient with HIV. Ganglioneuromas generally affect young children and are typically singular in nature. However, our patient was already 27 years old at the time of presentation, approximately three times the typical mean age at presentation of 7 years [3, 4]. In addition, he had evidence of metastasis to lymph nodes, which has seldom been reported in adult patients.

Most ganglioneuromas are discovered incidentally and are typically located in the posterior mediastinum, retroperitoneum, or adrenal glands [4, 7]. When symptoms are present, they are largely non-specific and can be because of mass effect or, more rarely, hormone secretion [4, 5, 7]. In this case, our patient’s only presenting symptom was abdominal pain, which made diagnosis particularly challenging as clinicians would not consider ganglioneuroma as a probable cause in his age group. Additional symptoms reported in the literature include visual or palpable mass, abdominal distention, hypertension, or claudication due to vessel impingement [5], none of which were seen in our patient.

The patient was HIV positive, which may have influenced his presentation. HIV patients are well known to be at increased risk of cancer due to oncogenic viral infection such as Kaposi’s sarcoma, non-Hodgkin lymphoma, and cervical cancer [8, 9]. Additionally, there is some evidence suggesting that those with HIV are at increased risk of a wide range of other cancers including those of the lungs and kidneys [8, 9]. There are no published studies specifically linking HIV to ganglioneuromas. Although there is no reason to suspect that the patient’s cancer was the direct result of HIV, it may have been a contributing factor.

While not specific, imaging provides a valuable tool in the workup of patients with ganglioneuromas. Imaging descriptions of ganglioneuromas are largely based on studies that include mostly children. On contrast-enhanced CT, ganglioneuromas typically appear as a homogeneous low attenuation mass with progressive but incomplete enhancement [10, 11]. Calcifications are present in the minority of cases [10, 11]. On MRI, the mass tends to be T1 hypointense and T2 hyperintense [10–12]. Ultrasound is not typically useful for ganglioneuromas as findings are non-specific [13]. The retroperitoneal mass is often well circumscribed on cross-sectional imaging with a propensity to encase vasculature and displace adjacent organs [11].

Diagnosis of ganglioneuroma requires analysis of tissue samples by excisional biopsy, core needle biopsy, or fine-needle aspiration [14]. Of the neuroblastic tumors, ganglioneuromas are the most well differentiated and have the best prognosis [4, 5, 13]. Expected histological findings for ganglioneuroma include mature ganglion and Schwann cells [7, 15, 16]. In our patient, pathologic analysis of the tumor revealed a Schwannian stroma dominant ganglioneuroma, maturing subtype, with favorable histology. Mitotic figures or neuroblasts are not expected; if present, these would suggest a different diagnosis, such as neuroblastoma [16]. Average tumor size on resection is about 8 cm in diameter, and while the tumor may appear to be encapsulated, a true capsule is rarely present [4].

Our patient was found to have metastatic ganglioneuroma in adjacent lymph nodes. This is a rare finding, as ganglioneuromas do not typically present with nodal spread beyond the primary mass. The most widely proposed cause for the presence of multiple tumors is independent development. In this theory, a malignant neuroblastic tumor (either neuroblastoma or ganglioneuroblastoma) is present in the patient first and metastasizes to distant locations. Once spread, the multifocal tumor may mature into a ganglioneuroma [5, 6]. This would give the appearance of metastasis at surgical staging and is the probable cause of tumor extension to lymph nodes in our patient.

While ganglioneuromas are benign, they may cause functional problems because of involvement of adjacent structures, necessitating surgical intervention to remove the mass. However, in cases like our patient’s, with extensive involvement of surrounding structures, complete resection may be contraindicated as damage to vital structures (neurovasculature) may cause significant postoperative morbidity such as bleeding, ischemia, or neurological dysfunction [17]. In such patients, the decision to undergo incomplete resection to lessen these risks is considered sufficient for treatment. Studies have shown that an incomplete resection leaving residual tumor less than 2 cm has a low rate of progression [18]. Long-term follow-up for tumor surveillance should continue to be performed in patients undergoing incomplete resection.

Ganglioneuromas have excellent prognosis and are unlikely to cause tumor-related death [19]. Rates of event-free survival and tumor progression between complete resection and resection with minor residuals (less than 2 cm) are not significantly different. However, resections leaving residuals of more than 2 cm may result in tumor progression and inferior event-free survival [18]. Complications in tumor resection are common and may occur in up to 30% of patients [20]. Most of these complications are often transient and will resolve over time with little to no lasting effects; however, persistent complications are possible and may affect the decision to perform surgical resection [20].

## Conclusion

Retroperitoneal ganglioneuromas occur most often in children but may rarely occur in adults. While generally limited to a solitary tumor, ganglioneuroma may occasionally have metastatic spread to local lymph nodes, as demonstrated by this case. The patient in this case was HIV positive; however, it is unclear if this is a contributing factor to this disease. Increased awareness of this rare presentation will improve patient care.

## Data Availability

Not applicable.

## Abbreviations

CT: Computed tomography
MRI: Magnetic resonance imaging
HIV: Human immunodeficiency virus
SMA: Superior mesenteric artery
H&E: Hematoxylin and eosin
